# Very Low Levels of 25-Hydroxyvitamin D Are Not Associated With Immunologic Changes or Clinical Outcome in South African Patients With HIV-Associated Cryptococcal Meningitis

**DOI:** 10.1093/cid/ciu349

**Published:** 2014-05-13

**Authors:** Joseph N. Jarvis, Tihana Bicanic, Angela Loyse, Graeme Meintjes, Louise Hogan, Chrissy H. Roberts, Shmuel Shoham, John R. Perfect, Nelesh P. Govender, Thomas S. Harrison

**Affiliations:** 1 Department of Clinical Research, Faculty of Infectious and Tropical Diseases, London School of Hygiene and Tropical Medicine, United Kingdom; 2 Botswana–University of Pennsylvania Partnership, Gaborone, Botswana; 3 Division of Infectious Diseases, Department of Medicine, Perelman School of Medicine, University of Pennsylvania, Philadelphia; 4 Research Centre for Infection and Immunity, Division of Clinical Sciences, St George's University of London, United Kingdom; 5 Institute of Infectious Disease and Molecular Medicine and Department of Medicine, University of Cape Town, South Africa; 6 Department of Medicine, Imperial College London, United Kingdom; 7 Transplant and Oncology Infectious Diseases Program, Johns Hopkins University School of Medicine, Baltimore, Maryland; 8 Department of Medicine, Division of Infectious Diseases, Duke University Medical Center, Durham, North Carolina; 9 National Institute for Communicable Diseases–Centre for Opportunistic, Tropical and Hospital Infections, National Health Laboratory Service and Faculty of Health Sciences, University of the Witwatersrand, Johannesburg, South Africa

**Keywords:** HIV, cryptococcal meningitis, vitamin D, tuberculosis, South Africa

## Abstract

***Background.*** Vitamin D deficiency is associated with impaired immune responses and increased susceptibility to a number of intracellular pathogens in individuals infected with human immunodeficiency virus (HIV). It is not known whether such an association exists with *Cryptococcus neoformans*.

***Methods.*** Levels of 25-hydroxyvitamin D (25[OH]D) were measured in 150 patients with cryptococcal meningitis (CM) and 150 HIV-infected controls in Cape Town, South Africa, and associations between vitamin D deficiency and CM were examined. The 25-hydroxyvitamin D levels and cryptococcal notifications were analyzed for evidence of reciprocal seasonality. Associations between 25(OH)D levels and disease severity, immune responses, and microbiological clearance were investigated in the patients with CM.

***Results.*** Vitamin D deficiency (plasma 25[OH]D ≤50 nmol/L) was present in 74% of patients. Vitamin D deficiency was not associated with CM (adjusted odds ratio, 0.93 [95% confidence interval, .6–1.6]; *P* = .796). Levels of 25(OH)D showed marked seasonality, but no reciprocal seasonality was seen in CM notifications. No significant associations were found between 25(OH)D levels and fungal burden or levels of tumor necrosis factor α, interferon γ, interleukin 6, soluble CD14, or neopterin in cerebrospinal fluid. Rates of fungal clearance did not vary according to vitamin D status.

***Conclusions.*** Vitamin D deficiency does not predispose to the development of CM, or lead to impaired immune responses or microbiological clearance in HIV-infected patients with CM.

## Abstract (precis)

Vitamin D deficiency may increase susceptibility to opportunistic infections in HIV-infected individuals. We found no evidence that vitamin D deficiency increases risk of cryptococcal meningitis or leads to impaired immune responses or microbiological clearance in HIV-infected patients with cryptococcal meningitis.

Cryptococcal meningitis (CM) is a leading cause of death in human immunodeficiency virus (HIV)–infected individuals in low-resource settings [1]. The causative organism, *Cryptococcus neoformans*, is a facultative intracellular pathogen that has developed numerous strategies allowing it to survive and replicate inside macrophages [2, 3]. Environmental exposure to *Cryptococcus* is universal [4]. In the context of impaired adaptive immune responses, the ability of *Cryptococcus* to evade macrophage killing leads to dissemination, disease, and ultimately death [5]. Although the primary immune defect leading to development of cryptococcal meningitis is impairment of CD4^+^ T-cell (CD4) responses, usually secondary to HIV infection [6], the effectiveness of macrophage recognition, processing, and killing of *Cryptococcus* is likely to play an important role in the evolution of infection [2, 3, 7].

Vitamin D is required for effective macrophage responses to a number of intracellular pathogens including *Mycobacterium tuberculosis* complex (MTB), where it plays a critical role in macrophage activation following Toll-like receptor (TLR) signaling, tumor necrosis factor alpha (TNF-α) release, interferon gamma (IFN-γ)–mediated cathelicidin function, phagolysosome maturation and autophagy, and intracellular killing of mycobacteria [8–13]. Macrophages from HIV-infected patients have particularly impaired antituberculous activity in the absence of adequate vitamin D levels [8, 14], consistent with the markedly increased susceptibility to tuberculosis during HIV infection [15].

Similar to tuberculosis, CM is caused by an inhaled pathogen that evades effective intracellular killing by alveolar macrophages, often establishes a latent infection in the lung, and disseminates and causes disease when effective T-cell–mediated immune responses are depleted in HIV infection [5]. Data show that HIV-infected patients who have had pulmonary tuberculosis are at increased risk of developing CM [16], raising the possibility of a shared immune defect over and above CD4^+^ T-cell depletion. We hypothesized that vitamin D deficiency may impair immune responses to *Cryptococcus*, leading to similar increases in susceptibility to disease and impairments of microbiological clearance to those seen in MTB infection.

To test this hypothesis, we performed a study in Cape Town, South Africa, consisting of 3 parts: (1) 25-hydroxyvitamin D (25[OH]D) levels were measured in patients presenting with CM and control patients with comparable CD4 counts drawn from the same population who did not have CM to determine whether vitamin D deficiency was associated with the development of CM; (2) 25(OH)D levels in the study population were analyzed for evidence of seasonality corresponding to sunshine hours, and Western Cape CM notifications from the South African National Institute for Communicable Diseases (NICD) covering the study period were analyzed for evidence of reciprocal seasonality; and (3) associations between 25(OH)D levels and disease severity, immune responses, and microbiological clearance rates were examined in patients with CM.

## METHODS

### Participants and Procedures

Participants were recruited at GF Jooste Hospital, Cape Town, South Africa, between July 2005 and May 2010. One hundred fifty participants were HIV-infected adults (aged ≥21 years) with a first episode of CM (cases), diagnosed by cerebrospinal fluid (CSF) India ink or cryptococcal antigen testing (titers ≥1:1024; Meridian Cryptococcal Latex Agglutination System, Meridian Bioscience, Cincinnati, Ohio), who were enrolled sequentially in 2 clinical trials examining different amphotericin B–based induction regimens [17, 18]. The studies were approved by the Research Ethics Committee of the University of Cape Town, and patients gave informed consent for blood and CSF samples to be used for research purposes. The component trials had the same inclusion and exclusion criteria, and have been described elsewhere [17, 18]. On study enrollment, history and clinical examination findings were recorded. Blood samples taken prior to antifungal therapy were used for plasma vitamin D quantification. Lumbar punctures (LPs) with quantitative CSF cultures were performed on days 1, 3, 7, and 14. Cryptococcal clearance (early fungicidal activity [EFA]) was calculated as the rate of decrease in log colony-forming units (CFU) per milliliter of CSF per day derived from the slope of the linear regression of log CFU per milliliter against time for each patient [19]. The CSF cell count and protein and glucose levels were determined. CSF interferon gamma (IFN-γ), tumor necrosis factor alpha (TNF-α), and interleukin 6 (IL-6) concentrations were measured in all patients using the Luminex multianalyte platform (Luminex) and Bio-Rad cytokine kits (Bio-Rad) [20]. CSF soluble CD14 (sCD14) and neopterin concentrations were measured for a subset of 90 sequential patients using Bio-Rad kits and manual enzyme-linked immunosorbent assay (ELItest Neopterin, BRAHMS Aktiengesellschaft, Hennigsdorf, Germany), respectively. Baseline CD4 cell counts were recorded for all patients. Patients were followed for 1 year and mortality outcomes recorded.

Recruited concurrently were 150 hospital-based control patients, who were HIV-infected adults (aged ≥21 years) with a nadir CD4 count ≤100 cells/µL and no current evidence of or prior history of cryptococcal disease, attending the hospital for management of either newly diagnosed HIV infection or an opportunistic infection other than CM. These patients were drawn from the same population as the cases during the same “risk period,” and would have been included as a case in the study had they developed CM. Basic demographic data, medical history, and current CD4 count were recorded, and a blood sample was taken for plasma vitamin D quantification. Among cases and controls, all patients currently taking antituberculosis medication with a clinical diagnosis of tuberculosis (both sputum acid-fast bacillus smear positive and smear negative) were defined as having active tuberculosis. Written informed consent was obtained from each control participant, and the study was approved by the Research Ethics Committee of the University of Cape Town.

### Vitamin D Levels

Plasma 25(OH)D concentrations were measured in stored baseline blood samples at St George's University of London using Immunodiagnostics Systems’ 25(OH)D kit (REF IS2700) on the iSYS multidiscipline autoanalyzer. Vitamin D status was defined according to standard criteria as normal (>75 nmol/L), insufficient (≤75 nmol/L), deficient (≤50 nmol/L), or severely deficient (≤25 nmol/L) [13, 21].

### Cryptococcal Meningitis Notifications

All incident laboratory-confirmed cases of cryptococcal disease from the Western Cape were reported to the NICD during the study period with date of specimen collection; surveillance audits were conducted to ensure complete reporting. A case of incident cryptococcosis was defined as the first episode of laboratory-confirmed disease in a patient (encapsulated yeasts observed by microscopic examination of an India ink–stained fluid, or a positive cryptococcal antigen test or culture of *Cryptococcus* species from any body site) diagnosed at a clinical laboratory in the Western Cape Province.

### Statistical Analysis

Data were analyzed using Stata version 12.0 (StataCorp, College Station, Texas), R version 3.0.2 (R foundation for Statistical Computing), and GraphPad Prism version 6 (Graphpad Software Inc, San Diego, California). Variables were compared across groups using unpaired *t* tests, 1-way analysis of variance, Kruskal-Wallis, χ^2^, or Fisher exact tests as appropriate. The 25(OH)D results were log transformed, geometric means and 95% confidence intervals (CIs) presented, and log-transformed results used in regression analyses. For the case-control analysis, crude and adjusted odds ratios (ORs) exploring the association between vitamin D deficiency and CM, and potential confounders in this relationship, were obtained using logistic regression analysis. Evidence for seasonality in 25(OH) D levels and cryptococcal case notifications was examined using Poisson regression models, which modeled monthly data using a general trend plus a sinusoidal wave for seasonal effect (cosinor regression modeling [22]). Assessment of seasonality was made by comparing the Akaike information criterion of models including or jointly omitting the sine and cosine terms using a likelihood ratio test. Among the CM cases, associations between 25(OH)D levels and disease severity at presentation, baseline CSF immune responses, rate of clearance of infection, and mortality were examined using linear and Cox regression modeling. Statistical significance was defined as *P* ≤ .05.

## RESULTS

Demographic and clinical characteristics of patients are summarized in Table 1. Patients with CM had a median CD4 count of 32 cells/µL, and severe disease at presentation, with high CSF fungal burdens (median, 5.3 [interquartile range, 4.3–5.8 log_10_ CFU/mL]) and a high proportion of altered mental status (13%). All CM patients were antiretroviral therapy (ART) naive. The control patients were similar to cases in terms of age and CD4 count, although a larger proportion was female, and 30% were already taking ART. Sixty-three (42%) control patients had a current diagnosis of tuberculosis, compared with 53 (35%) of the CM cases (*P* = .24). Thirty-four (23%) controls had advanced HIV infection alone, and the remaining 53 (35%) were being investigated or treated for opportunistic infections or complications of HIV infection (including 13 with gastroenteritis, 10 with pneumonia or bacterial sepsis, 6 with anemia, and 24 with other conditions including *Pneumocystis* pneumonia, Kaposi sarcoma, and candidiasis). All patients were black Africans.

**Table 1.** Patient Characteristics and 25-Hydroxyvitamin D Levels

Characteristic	CM Cases (n = 150)	Controls (n = 150)	Adjusted OR^a^	*P* Value
Age, y	32 (28–38)	32 (27–37)		.337
Male sex, % (No.)	41% (62)	17% (26)		<.001
CD4 count, cells/µL	32 (13–58)	40 (19–79)		.13
Active tuberculosis, % (No.)	35% (53)	42% (63)		.236
On ART, % (No.)	0% (0)	30% (45)		<.001
Duration of ART, d	…	55 (21–99)		…
Vitamin D, nmol/L^b^	38 (35–41)	36 (33–39)		.367
Vitamin D ≤75 nmol/L, % (No.)	93% (139)	95% (142)		.338
Vitamin D ≤50 nmol/L, % (No.)	75% (112)	72% (108)		.669
Vitamin D ≤25 nmol/L, % (No.)	18% (27)	26% (38)		.116
Fungal burden, log_1__0_ CFU/mL	5.3 (4.3–5.8)	…		…
Altered mental status, % (No.)	13% (19)	…		…
EFA, log_10_ CFU/mL/d	−0.52 (−0.39 to −0.69)	…		…
Mortality^c^, % (No.)	28% (41)	…		…
Vitamin D >50 nmol/L, % (No.)	25% (38)	28% (41)	1 (base)	.796
Vitamin D ≤50 nmol/L, % (No.)	75% (112)	72% (108)	0.93 (95% CI, .54–1.61)	

Data presented are median (interquartile range) or percentage (No.). Significance testing was performed using Kruskal-Wallis, χ^2^, or Student *t* test as appropriate.

Abbreviations: ART, antiretroviral therapy; CFU, colony-forming units; CI, confidence interval; CM, cryptococcal meningitis; EFA, early fungicidal activity; OR, odds ratio; vitamin D, 25-hydroxyvitamin D.

^a^ Variables that were associated with both case status and vitamin D deficiency with a *P* value ≤0.1 were considered to be potential confounders in the relationship between vitamin D deficiency and development of CM. The only variable meeting these criteria was season, which was adjusted for in the analysis reported here. Levels of 25-hydroxyvitamin D varied by season, with the highest levels in the first quarter of the year (mean, 48 nmol/L [95% CI, 43–52 nmol/L]), lower levels in the second quarter (mean, 33 nmol/L [95% CI, 29–38 nmol/L]), the lowest levels in the third quarter of the year (mean, 32 nmol/L [95% CI, 28–35 nmol/L]), and increasing levels in the fourth quarter (mean, 38 nmol/L [95% CI, 34–42 nmol/L]), analysis of variance *P* = .005. Further adjustment for sex, CD4 count, and ART status did not alter the findings (adjusted OR, 0.82 [95% CI, .44–1.51]; *P* = .523).

^b^ Log-normal distribution; geometric mean and 95% CIs are presented.

^c^ Mortality at 10 weeks.

### Vitamin D Deficiency Is Common, and Clear Seasonal Variations Are Observed

**Figure 1.**
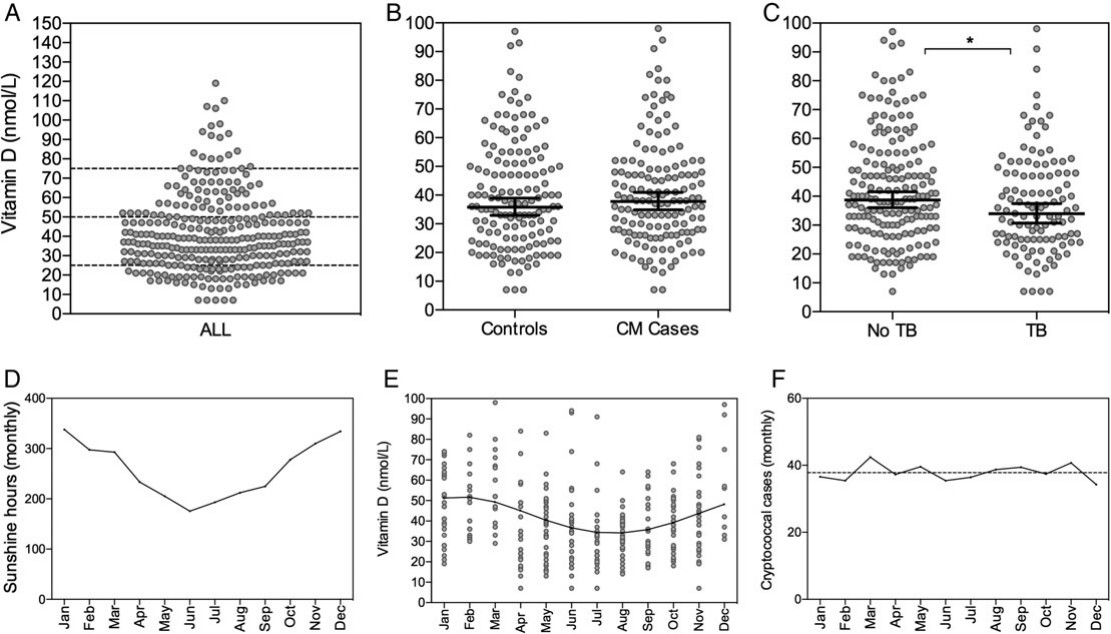
Plasma 25-hydroxyvitamin D (25[OH]D) levels by cryptococcal meningitis status, tuberculosis status, and season. *A*, Plasma 25(OH)D levels of the whole study population (cases and controls combined), with dashed lines at 75 nmol/L (vitamin D insufficiency), 50 nmol/L (vitamin D deficiency), and 25 nmol/L (severe vitamin D deficiency). *B* and *C*, Plasma 25(OH)D levels according to cryptococcal meningitis case status (*B*) and tuberculosis status (*C*), with lines at the geometric mean and error bars showing 95% confidence intervals. Levels of 25(OH)D were significantly lower in individuals with tuberculosis than in those without tuberculosis (*34 nmol/L vs 39 nmol/L; *P* = .029). *D*, Average number of sunshine hours per month in Cape Town (source: National Oceanic and Atmospheric Administration, available at: www.noaa.gov). *E*, Levels of 25(OH)D by month (averaged over the 5-year study period) with cosinor regression line. *F*, Monthly cryptococcal notification rates (averaged over the period 2005–2011) with best-fit regression line. Abbreviations: CM, cryptococcal meningitis; TB, tuberculosis.

The mean 25(OH)D concentration of the total study population (cases and controls combined) was 38 nmol/L (Figure 1). Only 18 (6%) had adequate 25(OH)D levels (>75 nmol/L). Two hundred twenty (74%) had vitamin D deficiency (≤50 nmol/L), and 65 (22%) were severely vitamin D deficient (≤25 nmol/L). Levels of 25(OH)D varied by season, with the highest levels in the first quarter of the year (mean, 48 nmol/L [95% CI, 43–52 nmol/L]), corresponding to the southern hemisphere summer and highest number of sunshine hours, and the lowest levels in the third quarter of the year (mean, 32 nmol/L [95% CI, 28–35 nmol/L]), corresponding to the winter months and lowest number of sunshine hours. Cosinor regression modeling confirmed the presence of significant seasonality in vitamin D levels (*P* < .001). The 25(OH)D levels did not differ by sex and were not associated with age, CD4 count, or ART status.

### No Seasonal Trends Are Evident in Cryptococcal Meningitis Notification Rates in the Western Cape Region

To examine associations between vitamin D status and the risk of developing CM, the Western Cape region CM notification rates for the 7-year period 2005–2011 were analyzed for seasonal trends. Despite the seasonal variation in 25(OH)D levels seen in this patient population, cosinor regression modeling did not demonstrate any seasonal trend in CM notification rates (*P* > .7), with an average of 39 cases per month during the period and very little monthly variation (Figure 1).

### Vitamin D Deficiency Is Not Associated With Cryptococcal Meningitis, but Is Associated With Active Tuberculosis

Levels of 25(OH)D levels did not differ between CM cases and control patients (mean, 38 nmol/L vs 36 nmol/L; *P* = .367; Table 1). Vitamin D deficiency was not associated with CM (OR, 1.12 [95% CI, .7–1.9]; *P* = .669), and this remained the case in a multivariable logistic regression model adjusted for season (adjusted OR [aOR], 0.93 [95% CI, .6–1.6]; *P* = .796). A sensitivity analysis restricted to ART-naive patients yielded the same findings (aOR, 0.82 [95% CI, .44–1.51]; *P* = .523), as did the equivalent analysis looking at severe vitamin D deficiency (aOR, 0.62 [95% CI, .32–1.31]; *P* = .223). Conversely, 25(OH)D levels were lower in patients with active tuberculosis compared with those without (34 nmol/L vs 39 nmol/L; *P* = .029), and this difference remained significant after adjusting for CM case status and CD4 count (*P* = .04). In both CM cases and controls, vitamin D deficiency was associated with increased odds of active tuberculosis, with some evidence for an increasing trend with worsening deficiency (OR, 1.47 [95% CI, .5–4.7] for vitamin D insufficiency; OR, 1.51 [95% CI, .5–4.5] for vitamin D deficiency; and OR, 2.52 [95% CI, .8–7.9] for severe vitamin D deficiency, all compared to a baseline of normal vitamin D status; *P* for trend = .069).

### Vitamin D Status Is Not Associated With Disease Severity, Host Immune Response, or Microbiological Clearance in Patients With HIV-Associated Cryptococcal Meningitis

**Figure 2.**
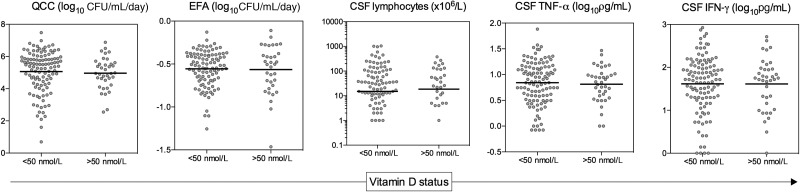
Fungal burden, cerebrospinal fluid (CSF) immune responses, and rate of clearance of infection in cryptococcal meningitis patients with and without vitamin D deficiency. The baseline CSF fungal burden (QCC), rate of clearance of infection (EFA), baseline CSF lymphocyte count, CSF TNF-α concentration, and CSF IFN-γ concentration are shown according to whether patients were vitamin D deficient (plasma 25-hydroxyvitamin D ≤50 nmol/L). Lines indicate the mean in the vitamin D–deficient patients and in those without vitamin D deficiency. No significant differences were present between the vitamin D–deficient and –sufficient groups in any of the variables shown. Abbreviations: CFU, colony-forming units; CSF, cerebrospinal fluid; EFA, early fungicidal activity; IFN, interferon; QCC, quantitative cryptococcal culture; TNF, tumor necrosis factor.

Among the 150 CM cases studied, there were no associations between 25(OH)D level and either fungal burden at disease presentation, the host immune response at the site of infection, or the rate of clearance of infection (Figure 2 and Table 2). Mean fungal burden was very similar in those with and without vitamin D deficiency (5.1 log_10_ CFU/mL vs 5.0 log_10_ CFU/mL; *P* = .687), as were CSF lymphocyte counts (15 × 10^6^/L vs 19 × 10^6^/L; *P* = .897), CSF TNF-α levels (0.84 log_10_ pg/mL vs 0.81 log_10_ pg/mL; *P* = .697), CSF IL-6 levels (2.44 log_10_ pg/mL vs 2.28 log_10_ pg/mL; *P* = .540), and CSF IFN-γ levels (1.62 log_10_ pg/mL vs 1.61 log_10_ pg/mL; *P* = .988). Regression modeling confirmed the absence of significant associations between 25(OH)D levels and fungal burden, CSF lymphocytes, CSF TNF-α levels, CSF IL-6 levels, and CSF IFN-γ levels (Table 2). Given evidence that in the context of tuberculosis infection the activation of macrophages by IFN-γ is vitamin D dependent [11], we examined the ratio of IFN-γ to the macrophage activation markers sCD14 and neopterin. The IFN-γ:sCD14 ratios (0.26 vs 0.25; *P* = .788) and IFN-γ:neopterin ratios (0.82 vs 0.83; *P* = .914) were similar in patients with and without vitamin D deficiency, providing no evidence for differential macrophage activation in CM patients according to vitamin D status.

**Table 2.** Associations Between Vitamin D Status and Fungal Burden, Immune Responses, and Rate of Clearance of Infection in Patients With Cryptococcal Meningitis

Variable	Vitamin D>50 nmol/L	Vitamin D≤50 nmol/L	*P* Value	β Coefficient^a^	*P* Value
Baseline fungal burden, log_10_ CFU/mL	5.0 (4.6–5.3)	5.1 (4.8–5.3)	.687	0.07 (−.32 to .47)	.702
CSF lymphocytes, ×10^6^/L^b^	19 (1–67)	15 (1–88)	.896	−39 (−93 to 14)	.148
CSF TNF-α, log_10_ pg/mL	0.81 (.70–.92)	0.84 (.76–0.92)	.697	−0.09 (−.21 to .03)	.148
CSF IFN-γ, log_10_ pg/mL	1.61 (1.41–1.81)	1.62 (1.49–1.74)	.988	−0.09 (−.30 to .11)	.374
CSF IL-6, log_10_ pg/mL	2.28 (1.84–2.72)	2.43 (2.19–2.69)	.540	−0.26 (−.68 to .17)	.231
CSF sCD14, log_10_ pg/mL	6.02 (5.91–6.11)	6.02 (5.97–6.09)	.834	0.03 (−.07 to .12)	.596
CSF neopterin, log_10_ pg/mL	1.85 (1.75–1.95)	1.90 (1.82–1.95)	.522	−0.05 (−.16 to .06)	.366
CSF IFN-γ:sCD14 ratio	0.25 (.21–.29)	0.26 (.23–.28)	.788	−0.02 (−.05 to .02)	.309
CSF IFN-γ:neopterin ratio	0.82 (.68–.97)	0.82 (.74–.90)	.914	−0.04 (−.17 to .08)	.497
Early fungicidal activity, log_10_ CFU/mL/d	−0.56 (−0.46 to −0.66)	−0.56 (−.51 to −0.60)	.847	−0.02 (−.09 to .06)	.701

Data are presented as means and 95% confidence intervals for the vitamin D–deficient and vitamin D–nondeficient groups.

Abbreviations: CFU, colony-forming units; CSF, cerebrospinal fluid; IFN, interferon; IL, interleukin; sCD14, soluble CD14; TNF, tumor necrosis factor; vitamin D, 25-hydroxyvitamin D.

^a^ The β coefficients are from linear regression analyses where the clinical and immunological parameters were considered individually as dependent variables, and 25-hydroxyvitamin D levels (log transformed) were considered as the explanatory variable. The coefficients shown thus represent the average increase in the dependent variable for each single unit increase (log_10_ nmol/L) in 25-hydroxyvitamin D concentration.

^b^ Heavily positively skewed; median values with interquartile ranges are shown.

In keeping with the absence of any observed impact of vitamin D status on the immune response to cryptococcal disease, rates of clearance of *Cryptococcus* from the CSF were not associated with 25(OH)D levels (β coefficient −0.015 [95% CI, −.09–.06]; *P* = .701). The mean rate of clearance was −0.56 in those with vitamin D deficiency vs −0.56 in those without (*P* = .847). Mortality at 10 weeks was 30% (n = 33) in patients with vitamin D deficiency vs 22% (n = 8) in those without (*P* = .367). After adjustment for CD4 count and the other key predictors of mortality, baseline fungal burden and abnormal mental status [23], the hazard of death was 1.35 (95% CI, .7–2.6; *P* = .375) in vitamin D–deficient patients compared with those non–vitamin D–deficient patients.

## DISCUSSION

Vitamin D deficiency was prevalent in this population of HIV-infected black African patients in Cape Town, consistent with previous findings in HIV-infected and uninfected populations in this setting, and, in keeping with previous reports, showed a marked seasonal variation closely related to sunshine exposure [14]. Also consistent with recent studies from Cape Town [14] was the observed association of vitamin D deficiency with active tuberculosis. We did not find any evidence for an association between vitamin D status and either susceptibility to CM or the immune response to CM and microbiological clearance in patients who had developed CM. Levels of 25(OH)D levels did not differ between the cohort of patients with CM and the control patients with comparable CD4 counts but no history of cryptococcal disease. This remained the case in sensitivity analysis adjusting for ART status, the only important factor differing between the CM cases and controls. Further evidence for an absence of association between vitamin D status and susceptibility to CM was the lack of seasonal trend in CM notifications, despite the clear seasonal variation in 25(OH)D levels in this population [14]. Consistent with these observations were our findings that vitamin D deficiency was not associated with fungal burden at CM presentation, did not influence the CSF immune response, and had no bearing on the rate at which infection was cleared from the CSF. As in prior studies [14], mean 25(OH)D levels in the studied population were low. Nevertheless, variation within a range of relatively low levels was associated with important differences in susceptibility to tuberculosis in this and other studies [14], arguing against the possibility that the lack of association seen with cryptococcal disease was due to low population vitamin D status.

Very few prior studies have examined vitamin D in the context of other fungal infections, and the reported results do not show a consistent association with vitamin D status, which may be related to the diverse host defense mechanisms involved [24, 25]. Our findings suggest that immune control and clearance of *Cryptococcus* is not via vitamin D–dependent pathways. Given the immunomodulatory effects of vitamin D on both innate and adaptive immunity [8–13, 26], plus reports demonstrating impaired immune responses and increased susceptibility to HIV and HIV-associated opportunistic infections such as tuberculosis, respiratory tract infections, and candidiasis [8, 9, 14, 26–28], the lack of any observed association with CM is perhaps surprising. The bulk of the data concerning the role of vitamin D in immunity to infectious diseases come from studies of tuberculosis. Convincing evidence shows that vitamin D deficiency is a risk factor for the development of tuberculosis [14, 27, 28], and data from controlled trials suggest that vitamin D replacement may improve outcomes in patients with tuberculosis [29]. Macrophages from vitamin D–deficient HIV-infected patients demonstrate impaired intracellular signalling and TNF-α expression in response to TLR2/4 signaling by MTB [8], and these responses are restored by vitamin D supplementation in vitro. Activation of MTB-infected macrophages by T-cell–derived IFN-γ is dependent on vitamin D [11], and can be restored in macrophages from vitamin D–deficient patients by vitamin D supplementation. Importantly, for restriction of MTB growth in macrophages, vitamin D promotes phagolysosome fusion and maturation [9, 11], the generation of reactive oxygen and nitrogen species [30, 31], production of antimicrobial cathelicidins [9, 11, 32], and induction of autophagy [9, 11]. These mechanisms overcome the immune evasion mechanisms employed by MTB of blocking phagosome maturation, and inhibiting phagosome-lysosome fusion [32–34]. In contrast to MTB, *Cryptococcus* does not need to prevent phagosome maturation or phagosome-lysosome fusion for intracellular survival, and is able to thrive in the acidic phagolysosome, protected by a thick polysaccharide capsule and virulence factors such as the ability to produce melanin using laccase, which protects against the oxidative burst [2, 3]. It is thus probable that vitamin D–dependent promotion of phagolysosome fusion and maturation has little effect on anticryptococcal immunity. Similarly, the promotion of cathelicidin production and autophagy, neither of which have a proven role in the innate response to cryptococcal infection [2], is unlikely to have significant anticryptococcal activity.

Activation of *Cryptococcus*-infected macrophages by T-cell–derived IFN-γ is likely to be critical for effective control of cryptococcal infection [35–37]. IFN-γ levels in the CSF are strongly associated with fungal burden and the rate of fungal clearance in patients with HIV-associated CM [20, 23], and exogenous IFN-γ has been shown to significantly increase the rate of clearance of cryptococci from the CSF [18]. Although we can only infer indirectly from our results, we found no evidence to suggest that IFN-γ–induced macrophage activation was vitamin D dependent, unlike in IFN-γ–induced activation of MTB-infected macrophages [11]. Levels of the macrophage activation markers sCD14 and neopterin, and the IFN-γ:sCD14 and IFN-γ:neopterin ratios did not differ according to vitamin D status.

Interestingly, there are limited data to suggest that the protective effects of vitamin D in the host response to MTB are due to anti-inflammatory properties, with inhibition of Th1-type immune responses [38, 39], faster resolution of inflammation [10], and limitation of the tissue damage associated with active MTB infection [26, 40]. Again in contrast to tuberculosis, tissue damage resulting from excessive inflammation is not a prominent feature of HIV-associated CM [41]. Rather, a lack of Th1-type inflammatory responses and high organism burdens are associated with poor outcomes in HIV-associated CM [18, 23, 37, 42], underlining the differing immune responses required for effective control of the opportunistic intracellular pathogens MTB and *Cryptococcus*.

In summary, we found no evidence that vitamin D deficiency predisposes to the development of CM, or leads to impaired immune responses or microbiological clearance in HIV-infected patients with CM. These data suggest that, in contrast to tuberculosis, vitamin D–dependent pathways are not of key importance in the host immune response to cryptococcal infection.
